# Dissecting social decision-making: A spotlight on oxytocinergic transmission

**DOI:** 10.3389/fnmol.2022.1061934

**Published:** 2022-12-22

**Authors:** Giulia Coccia, Filippo La Greca, Monica Di Luca, Diego Scheggia

**Affiliations:** Department of Pharmacological and Biomolecular Sciences, University of Milan, Milan, Italy

**Keywords:** social decision-making, oxytocin, brain circuit, empathy, prosocial

## Abstract

Social decision-making requires the ability to balance both the interests of the self and the interests of others to survive in social environments. Empathy is essential to the regulation of this type of interaction, and it often sustains relevant prosocial behaviors such as altruism and helping behavior. In the last decade, our capacity to assess affective and empathy-like behaviors in rodents has expanded our understanding of the neurobiological substrates that underly social decision-making processes such as prosocial behaviors. Within this context, oxytocinergic transmission is profoundly implicated in modulating some of the major components of social decision-making. Thus, this review will present evidence of the association between oxytocin and empathy-like and prosocial behaviors in nonhuman animals. Then, we will dissect the involvement of oxytocinergic transmission—across different brain regions and pathways—in some of the key elements of social decision-making such as emotional discrimination, social recognition, emotional contagion, social dominance, and social memory. Evidence of the modulatory role of oxytocin on social decision-making has raised considerable interest in its clinical relevance, therefore we will also discuss the controversial findings on intranasal oxytocin administration.

## Introduction

Survival in social environments is often complex and requires an intact functioning of the social decision-making ability that demands the right balance between the interests of the self and the interests of others. Surrounded by their conspecifics, individuals immersed in a society constantly relate to the social dimension and make decisions according to their mental states and intentions. Therefore, the equilibrium between self- and other-oriented aspects is fundamental for the ability to make appropriate social decisions and generate relevant prosocial behaviors such as helping behavior and altruism (Pfattheicher et al., 2022).

Empathy is described as an individual’s ability to understand and feel the emotions of others. It is often considered to be one of the main factors driving prosocial behaviors like altruism (Bartal et al., 2011; Decety et al., 2016; Scheggia and Papaleo, 2020), a behavior sometimes essential for survival in social environments (Rilling et al., 2008). Many non-human species have shown capabilities of empathy-driven prosocial behaviors towards their conspecifics (Decety et al., 2016; Scheggia and Papaleo, 2020), making them viable models for studying the neurobiological substrates of social decision-making processes (Scheggia et al., 2022). Along with empathy, social decision-making processes involving prosocial behaviors are affected by contextual information, such as familiarity (Scheggia et al., 2022), previous experiences (De Waal, 2008), stressors (Mudra Rakshasa and Tong, 2020), goals of interactions (Brucks and von Bayern, 2020), and individual differences (Bales and Perkeybile, 2012; Alonso et al., 2020).

Oxytocin is an evolutionarily conserved neuropeptide that modulates a large cluster of prosocial behaviors (Burkett et al., 2016; Yamagishi et al., 2020) and empathy-related processes (Pisansky et al., 2017). In a mammal’s brain, oxytocin is released by the paraventricular nucleus (PVN) and supraoptic nucleus (SON; Sofroniew, 1983; Landgraf and Neumann, 2004). The oxytocin receptor (OTR) is widely expressed in several brain regions and peripheral organs (Gimpl and Fahrenholz, 2001), modulating different functions and complex behaviors. The OTR is a member of the rhodopsin-type (class I) GPCR family, influencing gene expression, neuronal excitability, synaptic adaptation, and neurotransmission. OTR has been found in various types of neurons such as glutamatergic pyramidal cells, GABAergic interneurons, and neuroendocrine cells (Huber et al., 2005; Jurek et al., 2015; Lin et al., 2018). This neuropeptide modulates social abilities and social behaviors such as social recognition (Oettl et al., 2016), social preference (Dölen et al., 2013), social fear (Pisansky et al., 2017), emotional discrimination (Ferretti et al., 2019), and empathy-like (Burkett et al., 2016; Scheggia and Papaleo, 2020) and prosocial behaviors (Heinrichs and Gaab, 2007).

**Figure 1:**
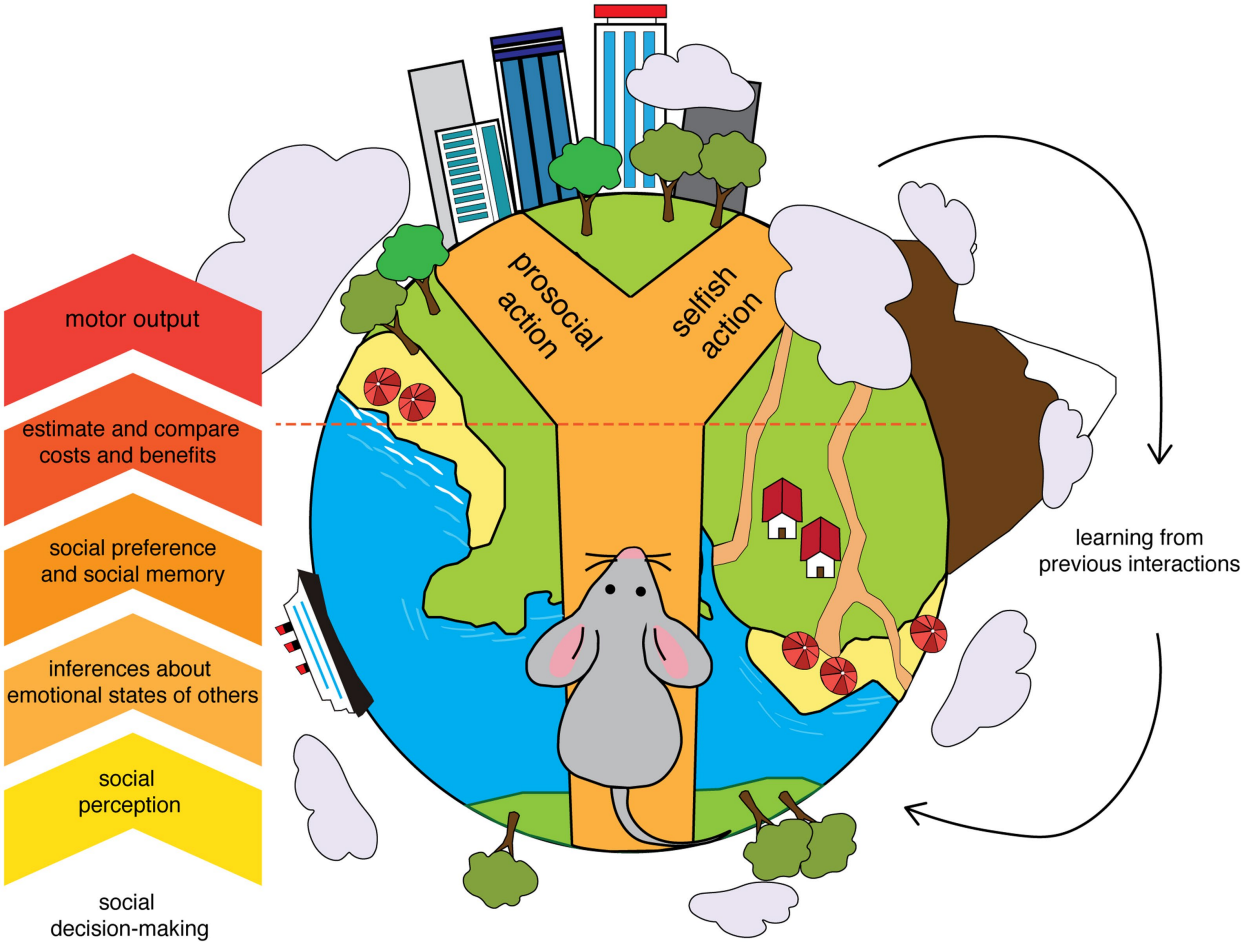
Core components of social decision-making. A simplified diagram showing some of the core components of the process of decision-making in a social environment. An anthropomorph mouse in the process of making a choice that could benefit others highlights how specific facets of this process are shared between rodents and humans. The diagram emphasizes the transformation from sensory information (social perception) to the motor output, that is a prosocial versus a selfish action. The interactions between the decision-making process and the consequences of the actions help to consolidate memories able to modulate future decisions.

Given the role of oxytocin in empathy-like and prosocial behaviors, our review offers an overview of the involvement of oxytocinergic transmission in the key components of social decision-making across different brain regions and pathways, such as emotional discrimination, emotional contagion, social dominance, and social memory (Figure 1). Finally, we will discuss the contrasting studies on the impact of oxytocin-based treatments in the clinical population.

### Oxytocin in social decision-making processes motivated by empathy-like behaviors

Some decisions and behaviors involving other individuals can be driven by empathy (Bartal et al., 2011; Burkett et al., 2016), while other kinds of prosocial behaviors, such as cooperation, are not necessarily related to empathy (Decety et al., 2016). The role of oxytocin in modulating empathy-driven behaviors—such as parental care and prosocial behaviors—has been largely explored in humans (Hurlemann et al., 2010; Guastella and Hickie, 2016) and rodents (Gur et al., 2014; Nakajima et al., 2014; Burkett et al., 2016). Marlin and colleagues found that, in mice, the oxytocinergic signaling in the left auditory cortex processes the behavioral response to a mouse pup’s distress calls and is also necessary for the maternal retrieval of isolated pups, enhancing the salience of vocal stimuli (Marlin et al., 2015).

Empathy can also motivate prosocial behaviors that are different from parental care. In a modified version of the human dictator game, in which one subject can choose between sharing or keeping the rewards from another participant, the infusion of oxytocin in the basolateral amygdala (BLA) of non-human primates favored the selection of prosocial tendencies (Chang et al., 2015). Oxytocin transmission in the insular cortex also mediates social decision-making in rats, modulating both approach and avoidance behaviors in a model of social affective preference (Rogers-Carter et al., 2018). Microinjections of an OTR antagonist in the insular cortex inhibited affective social behavior, with rats specifically avoiding juvenile stressed rats rather than stressed adults (Rogers-Carter et al., 2018). The oxytocin system is also involved in partner choice within groups of prairie voles. The intracerebroventricular infusion of a selective OTR antagonist prevented partner preference acquisition in mated male prairie voles, demonstrating the key role of oxytocin in pair bonding, which involves social decision-making processes (Johnson et al., 2016).

Several behavioral paradigms have been developed in the last decade aimed at investigating prosocial behaviors in rodents. It was found that rodents tend to approach and help stressed or trapped conspecifics (Bartal et al., 2011), avoid painful stimuli for the benefit of others (Hernandez-Lallement et al., 2020), and share food rewards with them (Scheggia et al., 2022), similarly to primates (Tan et al., 2017; Dal Monte et al., 2020). Yamagishi and colleagues found that the pharmacological block of the OTR in the anterior cingulate cortex (ACC) provoked a delay in learning helping behavior in rats, while the full acquisition of this behavior increased the activation of the immediate early gene c-Fos in ACC OTR-expressing neurons (Yamagishi et al., 2020). Another work from the same group revealed that OTR-knock-out prairie voles demonstrated impaired learning of the door-opening task and less interest in the soaked conspecific, suggesting that oxytocin modulates these helping and empathy-like behaviors in rodents (Kitano et al., 2022).

### Oxytocinergic transmission regulates key components of social decision-making

Social decisions require continuous interpretation of the surrounding context that also includes other social agents and their mental states and actions (Bartz et al., 2011). These decisions are multidimensional in nature, and involve cognitive and emotional facets related to both the self and others. These include emotional discrimination, emotional contagion, previous experiences, and other social factors such as group dynamics, where oxytocin transmission is profoundly implicated.

Identifying and recognizing a conspecific is crucial in making appropriate social decisions. This ability is influenced by intra- and inter-species differences, in which the perception of the conspecific varies widely. For example, while in primates the use of vision and gaze plays a critical role in social perception and cognition, rodents largely rely on olfactive information (Gangopadhyay et al., 2021). Oxytocin is involved from the beginning of the processes of decision-making, during which sensory information is extracted from the social context. In the olfactory system, oxytocin is required for social cue processing (Oettl et al., 2016). Perception of social cues is the basis of understanding others’ mental and emotional states. It has been reported that the PVN neurons projecting to the central amygdala (CeA) in mouse brains are selectively involved in emotional discrimination, likely through the interplay between oxytocin signaling and corticotropin-releasing factor, highly expressed in the CeA and involved in fear encoding (Ferretti et al., 2019). In addition, cortical areas are involved in the discrimination of conspecifics based on emotional states. Indeed, optogenetic inhibition of somatostatin-positive cells in the prelimbic region of the PFC (PL), which are highly enriched in OTR (Nakajima et al., 2014), impairs this ability (Scheggia et al., 2020).

Oxytocinergic signaling is also associated with mouse models of emotional contagion, a cognitive process by which observation of a conspecific in distress induces a similar affective experience in the observer. For example, both intranasal oxytocin administration (Pisansky et al., 2017; Zoratto et al., 2018) and chemogenetic stimulation of OTR-containing neurons (Pisansky et al., 2017) in mice increased the socially transmitted adoption of others’ emotional states, with a subsequent downregulation of OTR in the amygdala when oxytocin was chronically given (Pisansky et al., 2017). State matching with a familiar conspecific under stress can also motivate consolation behavior (Burkett et al., 2016), providing social buffering. This behavior was abolished by infusing an oxytocin receptor antagonist into the ACC. These reports highlight the relevance of oxytocinergic transmission in recognizing, understanding, and eventually sharing others’ emotions, which is often crucial for successfully navigating the social environment.

Social groups involve dominant and subordinate members, forming a hierarchy that can affect multiple behaviors (Qu et al., 2017). Therefore, hierarchies represent an important variable in social interactions and prosocial behaviors (Cronin, 2012). In both mice and rats, social dominance consistently promotes prosocial actions (Gachomba et al., 2022; Scheggia et al., 2022) similarly to non-human primates, showing that prosocial behaviors are more often directed towards downward ranks (Cronin, 2012). In this context, oxytocin reduces the differences in social behavior between dominant and subordinate members, thereby flattening the status hierarchy (Jiang and Platt, 2018). In line with this evidence, the oxytocinergic system underlying the establishment and maintenance of social hierarchies in rats (Timmer et al., 2011) and dominant mice shows increased OTR levels when compared with subordinate individuals (Lee et al., 2019).

The oxytocinergic network is sensitive to early-life stressors that can provoke long-term social impairments. He and colleagues showed that when mandarin voles— socially monogamous rodents with biparental attachment in their pups—were raised under paternal deprivation, they manifested anxiety-like behavior and lower social preference during adulthood (He et al., 2019). Importantly, the authors found that voles deprived of paternal influence had significantly fewer oxytocin neurons in the PVN and a decreased OTR in the medial PFC (mPFC) in both females and males, and optogenetic stimulation of PVN-to-PL projecting neurons restored this impairment (He et al., 2019).

The PVN oxytocin neurons also project to the anterior olfactory bulb, where OTR is largely expressed (Knobloch et al., 2012). Stimulating this pathway increased social recognition memory in female rats, while its inhibition prevented the ability (Oettl et al., 2016). Social memory is another important aspect with an influence on social decision-making processes. The role of the hippocampus in social behaviors has recently gained attention (Okuyama et al., 2016; for review, see Okuyama, 2018). In particular, the identification of the so-called “social place cells” in the dorsal hippocampus of bats (Omer et al., 2018) and rats (Danjo et al., 2018) points to this area as fundamental for processing self- and other-related information in the spatial dimension. The OTR is largely expressed across different subfields of the hippocampus (for review, see Cilz et al., 2019) and has an influence on social behaviors. It has been reported that the OTR conditional silencing selectively in CA2/CA3 or forebrain pyramidal neurons reduced the persistence of long-term social memory without affecting sociability or social novelty. In agreement with these findings, pharmacological stimulation of OTR on hippocampal slices provoked long-term potentiation (Lin et al., 2018). Intriguingly, Raam and colleagues showed that optogenetic inhibition of intrahippocampal connections between OTR-containing cells in dorsal CA2/CA3, projecting to the ventral CA1, impaired social but not non-social discrimination in mice (Raam et al., 2017). In a Magel^2tm1.1Mus^-deficient mouse, a model of autism-like disorders, an enhanced GABAergic activity of CA3 glutamatergic cells was found to be associated with an increased expression of OTR and somatostatin interneurons in both the dentate gyrus and CA2/CA3 regions. This effect might be responsible for their deficits in social memory, assessed using the social three-chamber task (Bertoni et al., 2021). Importantly, systemic administration of oxytocin in Magel^2tm1.1Mus^-deficient pups restored both hippocampal dysfunction and behavioral deficits during adulthood (Bertoni et al., 2021), highlighting the clinical relevance of oxytocin in the sphere of social cognition and behavior.

This evidence strongly supports the crucial role of oxytocin in modulating neural activity across several brain areas, recruited at different levels of the decision-making process, with significant effects on social and prosocial behaviors.

### Translating animal models: From endogenous system to oxytocin-based treatments

Fluctuations in endogenous oxytocin levels have been connected to both positive and negative modulations of social and prosocial behaviors (Crockford et al., 2014; Marsh et al., 2021; Tabak et al., 2022). This can be attributed to the influence of the oxytocin system on neural areas or circuitries related to reward (Scheele et al., 2013) and emotional processing, such as fear processing (Meyer-Lindenberg et al., 2011), attentional resources, and salience attribution to social stimuli (Dölen et al., 2013; Wei et al., 2022). Moreover, the anxiolytic effects driven by oxytocin changes can further contribute to the expression of social behaviors mainly due to the influence on the hypothalamic–pituitary–adrenal axis and the amygdala activity (Eckstein et al., 2015; Mitre et al., 2016; Neumann and Slattery, 2016). Furthermore, endogenous oxytocin levels are highly susceptible to sex, age, personality traits and predisposition, previous history, and context (Marsh et al., 2021). Genetics, epigenetics, and neurobiological factors have an impact on endogenous oxytocin, such as OTR variances (Rodrigues et al., 2009; Saphire-Bernstein et al., 2011; Spencer et al., 2022) and fluctuations across the lifespan (Audunsdottir and Quintana, 2022; Zak et al., 2022). These factors could modify the modulatory effects of oxytocin on social behaviors (Van IJzendoorn et al., 2011; Declerck et al., 2020; Marsh et al., 2021). For instance, contextual information associated with danger or social threat can stimulate oxytocin release that can be associated with aggressive-defensive or antisocial behaviors (Hurlemann and Marsh, 2019).

Dysregulation or malfunctioning of the oxytocin system has been reported in neuropsychiatric (Green et al., 2001; Yamasue and Domes, 2017; Goh et al., 2021) and neurodegenerative disorders (Gabery et al., 2015; Unti et al., 2018), mostly in the form of reduced endogenous oxytocin levels. In this case, the downregulation of the oxytocinergic transmission might be associated with anomalies in attention, evaluation, and response to external socio-emotional stimuli (Crockford et al., 2014; Gulliver et al., 2019). This could affect the cognitive and socio-emotional components necessary for expressing effective social decisions and behaviors regarding others (Marsh et al., 2021). Therefore, the assessment of endogenous oxytocin levels becomes crucial for the evaluation of the real effects of oxytocin-based treatments in the clinical setting (Marsh et al., 2021; Tabak et al., 2022).

Targeting oxytocinergic signaling has been considered to be an effective strategy to contrast social deficits in clinical populations. Intranasal application has been perhaps the principal route of oxytocin administration when compared with others, such as oral or intravenous, in the clinical and non-clinical setting (Quintana et al., 2021), due to the direct link with the central nervous system and the neglectable side-effects reported (Born et al., 2002; MacDonald et al., 2011; Bakermans-Kranenburg and van IJzendoorn, 2013). A good body of evidence revealed that the administration of intranasal oxytocin (IN-OXT) has beneficial properties on empathy and prosocial behaviors both in human (MacDonald and MacDonald, 2010; Geng et al., 2018; Leng and Leng, 2021) and nonhuman animals (Neumann et al., 2013; Huang et al., 2014; Chang et al., 2015; Pisansky et al., 2017; Zoratto et al., 2018). Specifically, studies on healthy human participants performing behavioral tasks readapted from the economic field (Sanfey, 2007) revealed that IN-OXT promotes and likely enhances relevant prosocial manifestations such as trust (Kosfeld et al., 2005), generosity (Domes et al., 2007), cooperation (De Dreu, 2012), altruism (Marsh et al., 2015), and social bonding (Lim and Young, 2006). However, subsequent efforts for replicating these initial results have failed, forcing researchers to downsize or review the claims around IN-OXT and its social properties (Nave et al., 2015; Declerck et al., 2020; Macchia et al., 2022).

Clinical studies reported beneficial effects on social dysfunctions following IN-OXT, including social decision-making deficits. Andari and colleagues reported increased trust and social preference within a virtual social interaction game involving 13 adult subjects with autistic spectrum disorder (ASD; Andari et al., 2010). More recently, clinical trials involving ASD participants revealed that long-term oxytocin-based treatments rescued neural activity or led to functional readaptations of areas such as the amygdala and the PFC, which are considered essential for establishing social interactions and making social decisions (Alaerts et al., 2020; Bernaerts et al., 2020). Nonetheless, Sikich et al. recently reported no effects after IN-OXT in measures of social functioning in a large-scale placebo-controlled study involving children and adolescents affected by ASD (Sikich et al., 2021). This was in line with a previous meta-analysis of 12 randomized clinical trials regarding the use of IN-OXT in ASD (Ooi et al., 2017).

In a meta-analysis by Bürkner and colleagues evaluating 12 randomized controlled trials in patients with schizophrenia, small but considerable effects of IN-OXT treatment on high-level social cognition were reported, including metallization and social inference abilities regarding others’ intentions and actions (Bürkner et al., 2017). Further, Wigton and colleagues observed a higher prosocial tendency after a single-dose oxytocin inhalation in 20 adult patients with schizophrenia during a rewarded decision-making task, likely due to better emotional and metallization capacities driving their decisions (Wigton et al., 2022). This improvement was linked to significant changes in the level of neural activity in key regions within the social decision-making system such as the amygdala, the ACC, and the insula (Wigton et al., 2022). The literature reports contrasting results, showing no significant benefits for social functioning in patients with schizophrenia following IN-OXT when compared with other treatments (Williams and Bürkner, 2017).

Administration of IN-OXT has also been applied in patients with neurodegenerative disorders that are characterized by social dysfunction, including social decision-making deficits (Manuel et al., 2020; Mason et al., 2021). Patients with frontotemporal dementia (FTD) who were administered a single dose of IN-OXT improved their abilities to recognize facial expressions (Jesso et al., 2011). Particularly, the authors indicated a reduced emotional response from FTD patients to negative faces. They also found a trend for better recognition of positive faces, which might lead to augmented trust and cooperative behavior within the social context (Jesso et al., 2011). Accordingly, Finger et al. used IN-OXT in a randomized clinical trial including 23 FTD patients and reported, indirectly, increased levels of empathy and social exchange in their relationships with their caregivers (Finger et al., 2015). Further, an ongoing trial is considering the long-term beneficial effects of IN-OXT for FTD patients (Finger et al., 2018). Finally, Labuschagne and colleagues described a significant recovery in Huntington’s disease patients’ ability to discriminate between emotions after oxytocin inhalation, a skill associated with restored neural activity in the areas involving emotion processing (Labuschagne et al., 2018). Despite this evidence, negative results have been reported on the effects of IN-OXT in neurodegenerative disorders. For instance, a recent meta-analysis did not find any improvement in emotion recognition or expression for the FTD population after IN-OXT treatment (Leppanen et al., 2017).

## Perspectives on future directions in clinical research

Although in many studies and clinical trials IN-OXT reduced social impairments, there is debate within the field about its effectiveness. As described, negative evidence exists for studies on neuropsychiatric (Dagani et al., 2016; Ooi et al., 2017; Williams and Bürkner, 2017; Sikich et al., 2021) and neurodegenerative (Leppanen et al., 2017) populations. Therefore, why is oxytocin in its current form not helpful for many patients? Preclinical studies should reduce the drift between basic and clinical research and translate knowledge into human applications. This process should not exist exclusively to create novel treatments, but also to adjust procedures of drug administration in patients. For instance, it has been shown that oxytocin increases the salience of social stimuli (Jurek and Meyer, 2020). This could suggest a pairing of oxytocin treatment with some type of behavioral training. Another crucial aspect of oxytocin administration is that it is still not clear how much of the dose is getting to the brain. A recent study developed a fluorescent sensor for real-time measurement of extracellular oxytocin. This could aid in the understanding of oxytocin destination administered by the intranasal route (Ino et al., 2022).

An important aspect to consider is that some of the oxytocinergic actions might also be mediated by vasopressin, which is structurally similar to oxytocin and comes from the same ancestral gene (Gwee et al., 2009; Theofanopoulou, 2021), with relevant effects on a large cluster of social behaviors and physiological functions (for review, see Song and Albers, 2018). Although oxytocin and vasopressin receptors are distributed differently across the brain, their interaction has several consequences (Xiao et al., 2017). Oxytocin can also bind to vasopressin receptors (V1aR), with antagonist effects on OTR (Anacker et al., 2016; Tan et al., 2019).

Finally, possible reasons for oxytocin failure could involve the way we measure its effects as well as problems in study design. The most relevant limitations include sample size (Gulliver et al., 2019; Marsh et al., 2021), individual (Declerck et al., 2020; Macchia et al., 2022) and contextual differences (Bartz et al., 2011), statistical inference and power (Calin-Jageman and Cumming, 2019; Mierop et al., 2020), and dosage characteristics (Kosaka et al., 2016). Furthermore, many studies lack the use of effective control groups or the comparison between oxytocin and other drug treatments (Erdozain and Penagarikano, 2020; Mierop et al., 2020). Likewise, the development of standardized procedures for measuring social abilities, including the components involved in social decision-making processes, might benefit oxytocin research in the clinical setting (Marsh et al., 2021; Tabak et al., 2022).

Thus, a more holistic and interactive approach regarding IN-OXT use in the clinical and nonclinical setting appears necessary (Audunsdottir and Quintana, 2022; Putnam and Chang, 2022). This would require an acknowledgment of the relationship between the exogenous ways of administration and the endogenous oxytocin (Mierop et al., 2020; Quintana and Guastella, 2020; Tabak et al., 2022). This expanded perspective also highlights the opportunity for an evaluation of the joint action between endogenous oxytocin and other neuropeptides like vasopressin (Rilling et al., 2012) or other neurotransmitters (Dölen et al., 2013). In addition, combinatorial treatments with other drugs (Fan et al., 2020) or additional behavioral and psychological techniques, which can exploit individual-contextual information (Marsh et al., 2021; Wei et al., 2022), should be considered.

## Concluding remarks

In recent years, considerable advances have been made in our ability to assess social decision-making processes in animal models. These advances had an impact on our understanding of the neurobiology underlying social decision-making processes, including when they take the form of prosocial behaviors that benefit others. Although not comprehensive, we reported increasing evidence of the modulatory role of oxytocin across the major elements in the process of making decisions in a social environment, from the perception of social stimuli to motor output. We highlighted a selection of studies and clinical trials that have reported the beneficial effects of oxytocin administration in neuropsychiatric conditions associated with dysfunctional social decision-making. However, given the heterogeneity of the responses to oxytocin-based treatments, we must address how we can best exploit our understanding of the oxytocin system through preclinical studies to target specific interventions for social dysfunctions in the clinical setting.

## Author contributions

GC is the main writer of the review and completed the collection and analysis of relevant literature. FG contributed to analysis of literature. DS contributed to conception of the study, edited the manuscript, and created the figure. GC and FG wrote the manuscript. ML reviewed the manuscript. All authors contributed to the article and approved the submitted version.

## Funding

This work was supported by funding from Fondazione Cariplo (2019-1747) to DS.

## Conflict of interest

The authors declare that the research was conducted in the absence of any commercial or financial relationships that could be construed as a potential conflict of interest.

## Publisher’s note

All claims expressed in this article are solely those of the authors and do not necessarily represent those of their affiliated organizations, or those of the publisher, the editors and the reviewers. Any product that may be evaluated in this article, or claim that may be made by its manufacturer, is not guaranteed or endorsed by the publisher.
